# Phytomedical assessment of two *Cymbopogon species* found in Nkonkobe Municipality: toxicological effect on human Chang liver cell line

**DOI:** 10.1186/s12906-017-1682-7

**Published:** 2017-06-02

**Authors:** Voster Muchenje, Beauty E. Omoruyi

**Affiliations:** 1 Department of Biochemistry and Microbiology, University of Fort Hare, Private Bag X1314, Alice, 5700 South Africa; 2 Department of Livestock and Pasture Science, University of Fort Hare, Private Bag X1314, Alice, 5700 South Africa

**Keywords:** Lemon plants, Essential oil, GC-ms, Chemical profiling, Antimicrobial activity, Toxicity evaluation

## Abstract

**Background:**

*Cymbopogon species* are widely used as herbal remedies by the traditional healers living in Nkonkobe Municipality for the treatment and management of skin and respiratory infections. According to our survey, the plants seem to be very important because of the higher demands.

**Methods:**

The leaves of *C. validis* and *C. plurinodis* were hydro-distilled and the resulted extracted oils were analyzed by GC/MS. Minimum inhibitory concentrations (MICs) ranging from 7.8 to 500.0 μg/ml of the extracted oils were tested against eight bacterial strains, using micro-well dilution method. The human Chang liver cell viability was determined using the CellTiter-Blue cell assay.

**Results:**

GC-MS analysis of the *C. validis* essential oil amounted to 87.03%, major components identified were Linalyl alcohol (18.9%), 2-Nephthalenemethanol (6.67%), Longifolene (6.53%), Cubedol (6.08%). Total oil percentage of *C. plurinodis* was 81.47% and the main components were characterized as 3-Cyclohexane-1-ol (13.58%), Nerolidol (13.6%) and 2-Carene (12.6%). The essential oils from both plants were found to be active against the growth of Gram positive than the Gram negative bacterial tested. Lethal dose at 50 (LD_50_) of both plants showed 74.87 ± 1.41 and 81.66 ± 1.40 degree of toxicity at 24 h.

**Conclusion:**

Both plants extracts were toxic to human Chang liver cell lines.

## Background

There are eight known species of *Cymbopogon* growing in the provinces of Mpumalanga, KwaZulu-Natal, Limpopo, Gauteng, North West, Eastern Cape and Western Cape. In addition, two others namely; *Cymbopogon plurinodis* and *C. validis* have been identified growing abundantly in the bushveld and pasture cultivated fields around Hosback area, in Nkonkobe Municipality, in the Eastern Cape. Morphologically, each of these plant species is quite different from each other, by the presence or absence of silica thones aligned on their leaf edges, leaves bear glandular hairs, and stacked with both basal and distal cells. Both plants are highly stress-tolerant plants which adapt easily to diverse climatic conditions [1].

According to the traditional healers of the study area, both plants can grow in all soil types. Cultivars of these plants prefer heavier soils such as loamy and gravely soil for quick growth, as this helps the plant to form dominant stands during dry seasons. The people of this region reported that these plants are effective against skin infection, sores, diabetes, infertility, high blood pressure and so on. Majority of them in the area are traditional healers (*Sangomas*) and rural dwellers, hence the use of medicinal plants for the treatment of certain diseases is very common. There is no doubt that some of these common diseases are usually caused by bacterial and viral pathogens, which definitely result to critical illness. *Bacillus cereus*, *Enterococcus faecalis*, *Listeria monocytogenes*, *Staphylococcus aureus*, *Escherichia coli*, *Klebsiella pneumonia*, *Salmonella typhimurium* and *Pseudomonas aeruginosa* are Gram-positive and negative bacteria and opportunistic pathogens of the human skin infection, toxic shock syndrome, urinary tract infections, gastroenteritis, and food poisoning [2].

*Cympbopogon validis* and *plurinodis* grow abundantly in this region and they are widely identified by the community dwellers as ‘Irwashu’ and ‘Unukayo’, respectively. As some of the rural dwellers are currently demanding for these plants because of their effectiveness, there is a dearth of information on their toxicity. Scientifically this study was therefore aimed at determining the plants’ phytomedical activities as well as evaluating its safety in an effort to validate their folkloric use in the treatment of microbial infections.

## Methods

### Plant extraction process

After obtaining the ethics certificate approved by the University of Fort Hare’s research ethics committee, the leaves of *Cymbopogon validis* and *Cymbopogon plurinodis* were collected in April, 2015 at 8 am in the morning in plastic bags. The botanical identification of these plants materials were confirmed by a botanist at the University of Fort Hare Institute. Voucher specimens were deposited at the institute’s Herbarium.

A hundred and eighty-eight grams (188 g) of each fresh leaves were hydro-distilled separately in a clevenger’s apparatus. Each samples were placed in a 5-L round bottom flask fitted to a condenser. After adding 4 L distilled water, the cooling condenser was connected with the distillation assembly and heated to boiling. After 30 to 40 min, boiling started; generated steam ruptured the cell walls of the leaves and released the oils present in the leaves. Distillation continued for 3 h for maximum oil recovery. The oil level in the separatory funnel was adjusted and maintained by varying the height of the outlet rubber tube. Once fixed, the excess water condensing in the seperatory funnel runs out spontaneously leaving accumulation of oil in the separatory funnel. After the distillation was over, each extracted oil was collected, filtered, and dried over anhydrous sodium sulphate (Na_2_SO_4_). For the determination of the procedure yield, the solvent was evaporated using a rotatory vacuum evaporator (R-114; Buchi, New Castle, USA). Final yield of both plants’ oil extracts were weighed and kept in separate clean bottles of known mass, labelled *C. validis* and *C. plurinodis*. The sensory characteristics of the essential oil from both plants were visualised based on their colour, clarity, aroma and odour intensity (Table 1). The yield obtained was calculated as follows: Mass of plant material distilled (g) = X; Mass of empty bottle (g) = A; Mass of bottle + oil (g) = B; Mass of oil (g) = (B-A); Percentage (%) yield = [(B-A) ÷ X] ×100. The resulting essential oils were stored at -20 °C prior to further analysis.

**Table 1:**
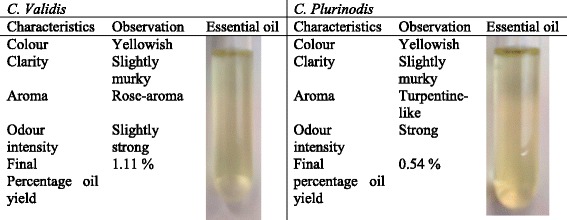
Sensory evaluation and final percentage oil yield of *Cymbopogon validis* and *Cymbopogon plurinodis*

### Phytochemical analysis

The phytochemical analysis of *C. validis* and *C. plurinodis* essential oil were determined by gas chromatography-mass spectroscopy instrument (HP 6890) with a mass selective detector (HP-5973). Identification of the chemical components of each essential oil was accomplished by marching their mass spectra and retention indices with those of the Wiley 275 library [3]. The quantity of compounds was calculated by integrating the peak areas of the spectrograms. A needle with 1.0 μl sample oil (essential oils tested) was inserted directly into the inlet of the Gas Chromatograph. The initial temperature 70 °C, maximum temperature 325 °C, equilibration time 3 min, ramp 4 °C/min, final temperature 240 °C; inlet: split less, initial temperature 220 °C, pressure 8.27 psi, purge flow 30 ml/min, purge time 0.20 min. Helium was used as a carrier gas at a flow rate of 8.27 psi; the mass spectrometer was operated at 70 eV, column capillary, 30 m × 0.25 mm ID fused silica column coated with DB-1: film thickness 0.25 μm, initial flow 0.7 ml/min, average velocity 32 cm/s; MS: El method at 70 eV. The scan time was 0.36 s with internal scan delay of 0.05 s and mass range 40–300. Compounds identified in the samples were confirmed by comparing their GC retention times with standards through a comparison of the mass spectra with available NIST Library 1 and with the softcopy results of the GC-MS Turbo Mass. Quantification of essential oil components, expressed in relative percentage on the total area of identified chromatogram, was carried out by peak area normalization measurements.

### Bacterial strains and chemicals

Four strains of Gram-positive bacteria: *Bacillus cereus* (ATCC #10702), *Enterococcus faecalis* (ATCC #29212), *Listeria monocytogenes* (ATCC #12022), *Staphylococcus aureus* (ATCC #6538) and four Gram-negative bacteria: *Escherichia coli* (ATCC #8739), *Klebsiella pneumonia* (ATCC #4354), *Salmonella typhimurium* (ATCC #13311) and *Pseudomonas aeroginosa* (ATCC #19582) were obtained from the Department of Biochemistry and Microbiology, University Fort Hare, Alice, South Africa. Ciprofloxacin, *p*-iodonitrotetrazolium violet (*p*-INT) and dimethylsulfoxide (DMSO) were purchased from Sigma-Aldrich (St. Louis, Missouri).

### Cell line growth and maintenance

The human Chang liver cell line used in this study was donated by Professor Maryna van de Venter from Nelson Mandela Metropolitan University, South Africa. Briefly, vials containing cells were taken from liquid nitrogen stocks and thawed in a water bath of approximately 37 °C, and then transferred to a 25 mm^3^ culture flask (TPP, Switzerland). A I ml thawed cell stock was diluted with 9 ml pre-warmed Dulbecco’s minimum essential medium (DMEM) containing 10% fetal bovine serum (FBS). The cells were incubated in a 37 °C humidified incubator (Shel Lab, USA), 5% CO_2_ for multiplication and adherence. Maintenance of cells was achieved by splitting the cells until the desired cell number and confluence was reached.

### Preparation of bacterial inoculums

The above bacteria strains were selected based on their public health disease-causing food borne poisons, bloody diarrhoea, anaemia, meningitis, pneumonia, etc. The inoculums of the test bacteria were prepared using the colony suspension method [4]. Colonies picked from 24 h-old cultures grown on nutrient agar were used to make suspensions of the test organisms in saline solution to an optical density of approximately 0.1 at 600 nm. The suspension was then diluted 1:100 *v*/v by transferring 0.1 ml of the bacteria suspension to 9,9 ml of sterile Mueller Hinton broth. The cell turbidity was assessed spectroscopically in comparison to that of the 0.5 McFarland standards (approximately 1.5 × 10^8^ cfu/ml) before being used for antibacterial assays [5].

### Antibacterial activity assay

**Fig. 1:**
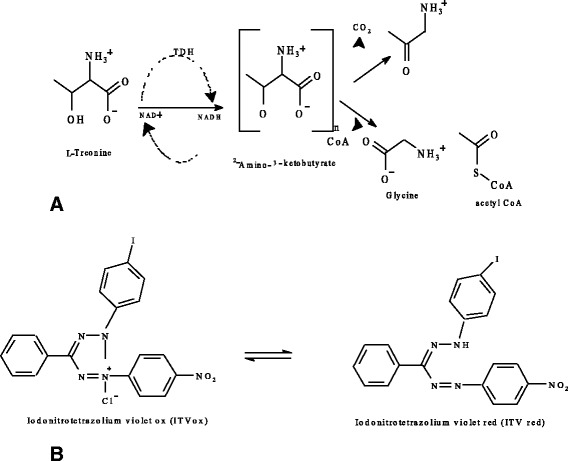
**a** Reaction pathways for the assay of threonine dehydrogenase, **b** INT, coupling reagent for the colorimetric assay

Antibacterial assay was determined using a micro-well dilution method [6]. Each plant’s essential oil was dissolved in DMSO and then in Mueller Hinton broth to reach a final concentration of 500.0 μg/ml. Two-fold serial dilutions were made in a concentration range from 7.8 to 500.0 μg/ml in sterile test tubes containing Mueller Hinton broth. The 96-well plates were prepared by dispensing into each well 95 μl of Mueller Hinton broth, 5 μl of the bacteria inoculum and a 100 μl from each serially diluted essential oil, transferred into six consecutive wells. The last well containing 195 μl of Mueller Hinton broth without compound and 5 μl of the inoculum on each strip was used as a negative control. The final volume in each well was 200 μl. Antibiotics of ciprofloxacin at same concentration range of 7.8 to 500.0 μg/ml was also prepared in Mueller Hinton broth and used as standard drug for positive control. Contents of each well were mixed on a plate shaker at 300 rpm for 20 s prior to incubation at 37 °C for 24 h. Each experiment was tested in triplicate. As an indicator of micro organism growth, 40 μl of *p*-iodonitrotetrazolium violet (*p*-INT) dissolved in water were added to the wells and incubated at 37 °C for 30 min [7]. The colourless tetrazolium dye acts as an electron acceptor and is reduced to a red-coloured formazan product by biologically active organisms (Fig. 1). Where microbial growth was inhibited, the solution in the well remained clear after incubation with INT and was taken as its minimum inhibitory concentration (MIC^INT^) at which no red colour occur. This was confirmed by plating 5 μl samples from clear wells on Mueller Hinton agar medium. The determinations of MIC values were done in triplicate.

### Cytotoxicity assay

Toxicological effect of both plant extracts were evaluated on human Chang liver cell lines using microculture CellTiter-Blue viability (Promega, USA) assay. Briefly, 96-well microplates were seeded accurately, with 100 μl DMEM + high glucose, L-glutamine and sodium pyruvate (Thermo Scientific, South Logan, Utah, USA) containing 3.0 × 10^3^ cells in suspension and incubated in a CO_2_ incubator regulated at 37 °C and 5% CO_2_. After 24 h incubation and attachment, the cells were treated with 1000, 500, 250, 125, 75, 25 and 5 μg/ml concentration of each extracts. Exactly 60 μm of curcumin (Sigma-Aldrich, South Africa) was used as positive control and 0.1% DMSO as negative control. After 24, 48, and 72 h of incubation, cell viability was determined by adding CellTiter-Blue as an indicator and further incubated for 4 h. Fluorescence was read at 570/620 nm using Analytical & Dignostic product Gen spectrophotometer (Bio Tek, USA).

## Results and discussion

### Sensory evaluation and essential oil yield

The first steps of plant essential oil testing usually begin with sensory evaluation. This help to evaluate the colour, clarity and odour intensity of any essential oil. These attributes are usually stored in their security cells, such as cavities, glandular trichomes or epidermic cells [8]. The results of the sensory evaluation and final percentage oil yield from *Cympbopogon validis* and *Cympbopogon plurinodis* is shown in Table 1.

Common characteristics observed from the essential oil extracts from both plants were yellowish in colour and slightly murky. The odour of *C. valids* was having a rose-like aroma and slightly strong, while that of *C. plurinodis* was turpentine-like, and very strong. Their final percentage of the essential oil yields were 0.54% and 1.11%, respectively.

### Chemical compounds of the essential oil

The Gas chromatography/mass spectroscopy analysis of the plants’ essential oils showed varied occurrence of volatile compounds which are mostly found in food and medicine. Hydro-distilled analysis of *C. validis* resulted in the identification of 80 phyto-compounds and the total amount of their calculated peak area percentage was 87.03% (Table 2). On the other hand, about 70 phytocompounds of *C. plurinodis* were identified and the total peak area percentage amounted to 81.47% (Table 3). The major compounds of *Cymbopogon validis* essential oil, based on the mass spectra peak areas were identified as Linalyl alcohol (18.9%), 2-Nephthalenemethanol (6.67%), Longifolene (6.53%), Cubedol (6.08%), β-Myrcene (4.85%), Santolina triene (4.60%), geraniol (2.68%), and 4-epi-cubedol (2.41%), while the main compounds of *Cymbopogon plurinodis* were characterized as 3-Cyclohexane-1-ol (13.58%), Nerolidol (13.6%), 2-Carene (12.6%), - Selinene (8.50%), β-Myrcene (4.73%) and D-Limonene (3.65%) (Tables 2 and 3).

**Table 2:** Chemical compounds of *Cymbopogon validis* leaf

Peak number	Phyto-compounds	Rt (mins)	Area %	Library quality match (%)	Chemical formulas
1	Cyclopentanol, 3-methylene	3.26	0.01	70	C_5_H_10_O
2	2-Hexenal	3.33	0.03	92	C_6_H_12_O
3	Trans-7-methyl-3-octene	3.40	0.01	72	C_9_H_18_
4	4-Heptanone	3.46	0.02	87	C_7_H_14_O
5	2-Heptanone	3.58	0.06	74	C_7_H_14_O
6	2-Heptanol	3.64	0.06	83	C_7_H_16_O
7	3-Carene	3.93	0.19	94	C_10_H_16_
8	α-Pinene	4.01	0.37	96	C_10_H_16_
9	Camphene	4.15	0.68	97	C_10_H_16_
10	β-Phellandrene	4.32	0.03	94	C_10_H_16_
11	β-Myrcene	4.41	4.85	86	C_10_H_16_
12	Octanal	4.49	0.01	86	C_8_H_16_O
13	α-Phellandrene	4.57	0.03	64	C_10_H_16_
14	Allylidenecyclohexane	4.61	0.05	45	C_9_H_14_
15	4-Carene	4.67	0.01	98	C_10_H_16_
16	Santolina triene	4.78	4.60	86	C_10_H_16_
17	β-Ocimene	4.87	2.72	94	C_10_H_16_
18	4-Methy-1,5-Heptadiene	4.92	0.11	64	n/a
19	γ-Terpinene	5.00	0.02	97	C_10_H_18_O
20	Hexanethioic acid	5.07	2.53	91	C_6_H_12_OS
21	Ethyl 2-(5-methyl-5-vinyltetrahydrofuran-2-yl)propan-2-yl carbonate	5.13	1.44	91	n/a
22	Linalyl alcohol	5.37	18.9	92	C_10_H_18_O
23	4-chlorobenzoic acid, 4-hexadecyl ester	5.45	0.11	90	C_23_H_37_ClO_2_
24	2,4,6-Octatriene	5.54	0.65	97	C_8_H_12_
25	5-Tetradecen-3-yne	5.63	0.07	72	C_14_H_24_
26	Citronellal	5.72	0.77	96	C_10_H_20_O
27	3-Hexadecene	5.82	0.09	83	C_16_H_32_
28	Endo-Borneol	5.92	0.69	97	C_10_H_18_O
29	Terpinen-4-ol	5.99	0.08	96	C_10_H_18_O
30	α-Terpineol	6.07	0.59	87	C_10_H_18_O
31	Disparlure	6.20	0.03	64	C_19_H_38_O
32	Citronellol	6.26	1.47	98	C_10_H_18_O
33	2,6-Octadien-1-ol, 3,7-dimethylene	6.33	0.04	86	C_10_H_16_O
34	Citral	6.40	0.16	95	C_10_H_16_O
35	Geraniol	6.48	2.68	95	C_10_H_18_O
36	4-Undecanone	6.60	0.94	95	C_11_H_22_O
37	Trans-Geranylgeraniol	6.74	0.01	68	C_20_H_34_O
38	Bornyl acetate	6.78	0.06	99	C_12_H_20_O_2_
39	*d*-Nonalactone	6.85	0.05	38	C_9_H_16_O_2_
40	2-Methoxy-4-vinylphenol	6.95	0.03	38	C_9_H_10_O_2_
41	1-Ethynylcyclopentanol	7.05	0.01	64	C_7_H_10_O
42	2,6-Octadiene,2,6-dimethylene	7.14	0.99	97	C_10_H_16_
43	α-Cubebene	7.24	0.11	99	C_15_H_24_
44	Geranyl acetate	7.35	1.97	90	C_12_H_20_O_2_
45	Epizonarene	7.42	0.31	98	C_15_H_24_
46	Cyclohexane, 1-ethenyl-1-methyl-2,4-bis-1-methylethenyl.	7.54	1.26	99	n/a
47	Formic acid	7.65	0.04	55	CH_2_O_2_
48	Caryophyllene	7.79	0.64	98	C_15_H_24_
49	α-Cubebene	7.97	0.81	70	C_15_H_24_
50	Epi-Bicyclosesquiphellandrene	8.05	0.05	87	C_15_H_24_
51	γ-Muurolene	8.10	0.65	83	C_15_H_24_
52	α-Muurolene	8.14	1.66	99	C_15_H_24_
53	4-epi-cubedol	8.52	2.41	93	C_15_H_26_O
54	Nephthalene	8.31	0.96	96	C_10_H_8_
55	Cubedol	8.39	6.08	95	C_15_H_26_O
56	Di-epi-alpha-cedrene	8.47	0.57	87	n/a
57	Guaia-1,11-diene	8.56	2.05	46	C_15_H_24_
58	Epizonarene	8.61	0.22	81	C_15_H_24_
59	Cyclohexane	8.64	0.44	30	C_6_H_12_
60	Gleenol	8.77	0.54	97	C_15_H_26_O
61	2-Naphthalenemethanol	8.96	6.67	86	C_11_H_10_O
62	Longifolene	9.08	6.53	93	C_15_H_24_
63	Hinesol	9.19	2.14	93	C_15_H_26_O
64	Agarospirol	9.25	0.37	98	C_15_H_26_O
65	Alloaromadendrene	9.42	0.37	53	C_15_H_24_
66	Isolongifolene	9.53	0.46	38	C_15_H_24_
67	Ethyladamantane-1-carboxylate	9.70	0.19	38	C_13_H_20_O_2_
68	β-Humulene	9.77	0.12	44	C_15_H_24_
69	Isocaryophillene	9.87	0.12	56	C_15_H_24_
70	Ledol	10.0	0.05	51	C_15_H_26_O
71	Acetic acid	10.1	2.69	38	C_2_H_4_O_2_
72	α-Guaiene	10.2	0.03	52	C_15_H_24_
73	4-Fluorobenzoic acid	10.4	0.02	62	C_7_H_5_FO_2_
74	Octasiloxane	11.3	0.01	41	H_6_OSi2
75	2-Nonadecanone	12.2	0.01	64	C_19_H_38_O
76	Hexamethylene	13.2	0.01	43	C_7_H_14_
77	Arsenous acid	13.8	0.04	43	H_3_AsO_3_
78	Diethyl ether	14.2	0.05	38	C_4_H_10_O
79	3-hexenyl ester	14.6	0.02	38	C_10_H_16_
80	4- hexadecyl ester	14.8	0.11	43	C_16_H_33_
Total amount of compounds		87.03			
Monoterpene hydrocarbon		16.74			
Oxygenated monoterpenes		31.43			
Sesquiterpene hydrocarbon		14.26			
Oxygenated sesquiterpene		11.6			
Oxygenated diterpene		0.01			
Aldehydes		6.81			
Fatty acids		2.78			
Others		3.40

**Table 3:** Chemical compounds of *Cymbopogon plurinodis* leaf

GC peak number	Phyto-compounds	Rt (mins)	Area %	Library quality match (%)	Chemical formulas
1	2-Hexenal	3.33	0.02	98	C_6_H_12_O
2	Heptanal	3.68	0.01	95	C_7_H_14_O
3	3-Carene	3.93	0.02	94	C_10_H_16_
4	α-Pinene	4.01	0.44	94	C_10_H_16_
5	Camphene	4.15	0.65	96	C_10_H_16_
6	Benzaldehyde	4.22	0.02	95	C_7_H_6_O
7	β-Phellandrene	4.32	0.05	87	C_10_H_16_
8	β-Myrcene	4.42	4.73	91	C_10_H_16_
9	2-Carene	4.56	12.6	94	C_10_H_16_
10	(+)-4-Carene	4.68	0.18	96	C_10_H_16_
11	D-Limonene	4.78	3.65	97	C_10_H_16_
12	β-Ocimene	4.86	0.05	97	C_10_H_16_
13	1-Octanol	5.01	0.08	68	C_8_H_18_O
14	4-Nonanol	5.05	0.25	83	C_9_H_20_O
15	6-Undecanol	5.17	0.11	53	C_11_H_24_O
16	Linalyl acetate	5.28	1.02	97	C_12_H_20_O_2_
17	Carveol	5.44	0.01	42	C_10_H_16_O
18	2-Cyclohexen-1-ol	5.67	1.19	95	C_5_H_10_O
19	3-Tetradecyn-1-ol	5.73	0.02	51	C_14_H_26_O
20	Camphor	5.76	0.01	94	C_10_H_16_O
21	Geraniol	5.82	0.05	93	C_10_H_18_O
22	1,3,5-Cycloheptadiene	5.88	0.16	53	C_7_H_10_
23	endo-Borneol	5.91	0.05	95	C_10_H_18_O
24	Benzenamine,3-ethoxy	5.96	0.14	43	C_6_H_7_NO
25	α-Terpineol	6.07	1.38	87	C_10_H_18_O
26	N-(2-Methyl-propenyl)-pyrrolidin-2-one	6.12	1.63	43	n/a
27	Photocitral B	6.46	0.01	49	C_10_H_16_O
28	3-Cyclohexen-1-ol	6.64	13.58	97	C_6_H_10_O
29	Bornyl acetate	6.79	0.24	98	C_12_H_20_O_2_
30	Hexanoic acid	6.84	0.06	52	C_6_H_12_O_2_
31	Furan	6.98	0.03	52	C_4_H_4_O
32	9-Hexadecenoic acid	7.06	0.01	35	C_16_H_30_O_2_
33	α-Ionone	7.19	0.48	91	C_13_H_20_O
34	3-Nonen-1-ol	7.27	0.04	87	C_9_H_18_O
35	Geranyl acetate	7.34	0.02	91	C_12_H_20_O_2_
36	Epizonarene	7.40	0.07	95	C_15_H_24_
37	Cycloheptane, 4-methylene-1-methyl-2-(2-methyl-1-propen-1-vinyl	7.49	0.10	50	n/a
38	Nephthalene	7.62	0.12	78	C_10_H_8_
39	Caryophyllene	7.80	1.32	99	C_15_H_24_
40	α-Guaiene	7.85	0.31	38	C_15_H_24_
41	Isoledene	7.93	0.10	97	C_15_H_24_
42	2,3-Octanedione	7.97	0.23	58	C_8_H_14_O_2_
43	Humulene	8.01	0.29	97	C_15_H_24_
44	β-Coapaene	8.06	0.17	93	C_15_H_24_
45	Alloaromadendrene	8.10	0.75	80	C_15_H_24_
46	4-epi-cubedol	8.38	2.22	99	C_15_H_26_O
47	γ-Muurolene	8.47	0.95	43	C_15_H_24_
48	Cycloprop (e) azulene	8.50	0.83	70	C_10_H_8_
49	Nerolidol 2	8.60	13.6	91	C_15_H_26_O
50	Trifluoroacetyl-α- fenchol	8.79	0.94	87	n/a
51	Caryophyllene oxide	8.87	0.83	94	C_15_H_24_O
52	Ethanopentalen-4-ol	9.09	0.90	38	n/a
53	Tau-Cadinol	9.13	0.76	86	C_15_H_26_O
54	Tau-Muurolol	9.25	1.94	62	n/a
55	- Selinene	9.34	8.50	83	C_15_H_24_
56	Trans- α-Bergamotene	9.43	0.73	87	C_15_H_24_
57	Pyrazole	9.68	1.59	43	C_3_H_4_N_2_
58	β-Pinene	9.76	0.37	58	C_10_H_16_
59	β-Santalol	9.82	0.38	53	n/a
60	Isoaromadendrene epoxide	10.0	0.02	91	C_15_H_24_O
61	Phytol acetate	10.0	0.05	80	C_22_H_42_O_2_
62	Epiglobulol	10.1	0.09	41	C_20_H_40_O
63	Diethyl ether	10.2	0.01	18	C_4_H_10_O
64	Shizukanolide	11.2	0.23	44	C_15_H_18_O_2_
65	2-Ethylacridine	11.4	0.01	25	C_15_H_13_N
66	Heptasiloxane	11.5	0.00	38	C_16_H_48_O_6_Si_7_
67	Ester	12.2	0.01	38	C_4_H_10_O
68	Octasiloxane	13.0	0.00	87	H_6_OSi2
69	Cyclotrisiloxane	13.2	0.01	43	C_6_H_18_O_3_Si_3_
70	1,3,5-Hexatriene	13.5	0.05	51	C_6_H_8_
Total of compounds		81.47			
Monoterpene hydrocarbon		25.49			
Oxygenated monoterpenes		18.37			
Sesquiterpene hydrocarbon		13.20			
Oxygenated sesquiterpene		17.66			
Oxygenated diterpene		0.14			
Aldehydes		0.49			
Fatty acids		0.09			
Others		6.03			

Generally, the phyto-constituents present in any plant essential oil are differentiated by their primary chemical groups of terpenic hydrocarbons, such as monoterpenes, sequiterpenes, diterpenes, aldehydes, esters, phenols, ketones and alcohols. All terpenes are essential building blocks in plant biochemistry and majority of them are used for different purposes in the pharmaceuticals, cosmetics, and food preservatives [9]. Some major components of terpene hydrocarbons such as, Carvcrol (4.1%), ɤ-Terpinene (39.26%) and Thymol (25.16%) from the essential oil of *Satureja thymbra* were found to have potent antibacterial inhibitory activity against all strains tested [10, 11]. In this study, the various chemical compounds from the essential oil of *C. validis* and *C. plurinodis* were profiled as monoterpenes (C_10_), oxygenated monoterpenes (C_10_O), sesquiterpenes (C_15_), oxygenated sesquiterpenes (C_15_O), diterpenes (C_20_), oxygenated diterpenes (C_20_O), aldehydes (CHO) and fatty acids (COH). From the chemical formulas of each *C. validis* phytocompounds in Table 2, the total amount of C_10_ monoterpenes hydrocarbons was 25.49%. Highest peak library match of compounds were β-Myrcene (4.85%) and Santolina triene (4.60%). On the hand, the total amount of *C. plurinodis* C_10_ monoterpenes hydrocarbons resulted to 16.74%. Highest peak values were 2-Carene (12.6%), β- Myrcene (4.73%) and D-Limonene (3.65%) (Table 3).

The total amount of C_10_O hydrocarbons which signifies the oxygenated monoterpenes was calculated to be 18.37% for *C. validis* essential oil. For *C. plurinodis* essential oil, the total amount of oxygenated monoterpenes was found lower with 31.43%. Linalyl alcohol (18.9%) and 3-Cyclohexane-1-ol (13.58%) were their highest peak match, respectively (Tables 2 and 3). The inhibitory activities of the essential oil extracted from *Ridolfia segetum*, *Oenanthe crocata* and *Santolina chamaecyparissus*, with their chemical constituents comprising of β-Myrcene, Santolina triene and Limonene have been reported active against HIV-1 reverse transcriptase, human tumor, oxidant and inflammatory activities [12, 13]. Limonene itself has immune-stimulatory, analgesic and anaesthetic properties [14]. Immune modulation and anti-proliferative effects of limonene’s anti-cancer activity have also been reported [14]. Manufacturing industries use 3-Cyclohexane and β-Myrcene as additives in producing perfumes, pesticides, polyvinyl, and nitrocellulose resins [15]. Over 200 families of Lamiaceae plants are known to produce large amounts of chemicals of Linalyl alcohol and 2-Nephthalenol, which are used as a scent in 60–80% of perfumed hygiene products, oxidizing colouring agents and cleaning agents including soaps, detergents, shampoos and lotions [16]. Medically, Linalyl alcohol therapy has been studied to reduced serum cortisol and improved the coronary flow velocity reserve (CFVR) in healthy men [17]. The findings revealed that Linalyl alcohol has a relaxation activity to relieve back pain, muscle stiffness, and cramps [17]. Another common downstream product of Linalyl alcohol is Vitamin E, a rich compound that naturally reduces cholesterol and the risk of developing cancer [18].

The total amount of C_15_ sesquiterpene hydrocarbons of *C. validis* essential oil gave 13.20%, compared to *C. plurinodis* sesquiterpene hydrocarbons, which gave 14.26%. Longifolene (6.53%) and -Selinene (8.50%) were found to be their highest peak match, respectively. The calculated total amount of *C. plurinodis* oxygenated sesquiterpenes was 11.6%, while that of *C. validis* was 17.66%. Nerolidol 2 (13.6%) and 4-epi-cubedol (2.41%) were observed as their highest values respectively. Essential oil containing sesquiterpenes are used to treat inflammatory and allergic infections [19]. Research has found that people who consistently use essential oil containing sesquiterpenes, have a higher level of resistance to illness than the average person. Further investigation revealed that if such an individual peradventure falls ill, he or she showed a 60–70% recovery than those not using essential oil products [20, 21]. Longifolene and -Selinene are some of the most abundant sesquiterpene hydrocarbons naturally occurring in *P. longifolia*, *P. roxburghii* and *P. sylyestris*. They are used as chemicals in perfumery industry owing to the woody odour of their chemically modified forms [22]. Both compounds have potent antioxidant and anti-inflammatory and anti-cancer properties. They offer assistance to a variety of metabolic and health problems, helps in weight management, liver detoxification enzymes, improve indigestion and sluggish bowel [23]. 4-epi-cubedol and Nerolidol are mainly found in some specific plants such as neroli, ginger, jasmine, lavender, tea tree, and lemon grass. They are mainly used as a food-flavoring agent and perfumery [24]. These compounds are currently under testing as a skin penetration enhancer for the transdermal delivery of therapeutic drugs [25]. Nerolidol works as a specific compound that female mites use to attract males for mating, in order words, Nerolidol is considered safe for humans and the environment.

Trans-geranylgeraniol (0.01%) Phytol (0.05%) and Epiglobulol (0.09%) were the major concentrated oxygenated diterpenes (C_20_O) detected in both plants essential oil of *C. validis* and *C. plurinodis*. These oxygenated compounds have the ability to inhibit microbial causing infections [26]. Phytol administered to mice at increasing dose responds of 25, 50, 10, and 200 mg/kg showed pronounced anti-nociceptive effects in the nociception models used [27, 28]. In vitro antioxidant activity of phytol demonstrated a strong effect against hydroxyl radicals and nitric oxide [27, 28]. A total content of aldehydes (CHO) present in *C. validis* was 6.81%. Of these, Nephthalenemethanol content showed the highest peak area of 6.67%. In contrast, 4-Nonanol (0.25%) was the highest peak value found in *C. plurinodis* aldehydes, from a total content of 0.49%. Fatty acids (COH) and their methyl esters present in both plant oils were present in smaller quantities, having total percentage of 0.09 and 2.78%, respectively. Medicinally, these compounds are known to have anti-inflammatory, anticancer, anti-amoebic, allelopathic, free radical scavenging and other useful biological activities [29].

The antibacterial activities of *Cymbopogon validis* and *Cymbopogon plurinodis* essential oils were assayed in vitro by a broth micro-dilution method against eight pathogenic bacteria strains. According to the results, *Cympbopogon validis* essential oil was found to be active against all the pathogenic bacteria, except *Klebsiella pneumonia* and *Pseudomonas aeroginosa* which demonstrated weak inhibitory activity at the highest MIC concentration of 500 μg/ml (Table 4). The strongest antibacterial inhibitory activity was seen against *Bacillus cereus*, *Listeria monocytogenes*, and *Staphylococcus aureus* with MIC values of 15.6 μg/ml followed by *Enterococcus faecalis* and *Escherichia coli* MIC 62.5 μg/ml, and then *Salmonella typhimurium* MIC at 125 μg/ml. In contrast, the essential oil of *Cympbopogon plurinodis* also displayed inhibitory activity against all the bacteria except *Pseudomonas aeroginosa*. Significant growth reduction was observed only against *Listeria monocytogenes* with MIC value of 15.6 μg/ml, followed by *Enterococcus faecalis* MIC 31.2 μg/ml, *Bacillus cereus* at 62.5 μg/ml, *Staphylococcus aureus* at 125.0 μg/ml and *Salmonella typhimurium* at 250 μg/ml. Weak MIC inhibitory activity of 500 μg/ml was observed for *Escherichia coli* and *Klebsiella pneumonia*. The standard antibiotic ciprofloxacin showed potent inhibitory action against all bacteria tested. At the lowest MIC of 7.8 μg/ml, ciprofloxacin was observed to reduce the growth of *Bacillus cereus* and *Listeria monocytogenes*, followed by *Staphylococcus aureus*, *Escherichia coli* and *Salmonella typhimurium* at MIC value of 15.6 μg/ml. Ciprofloxacin MIC activity on *Klebsiella pneumonia* was 31.2 μg/ml, *Enterococcus faecalis* at 62.5 μg/ml and *Pseudomonas aeroginosa* at 125.0 μg/ml.

**Table 4:** Antibacterial activity of *Cympbopogon validis* and *Cymbopogon plurinodis* essential oils

Microorganisms	Gram+/−	MIC^INT^ values (μg/ml)		Ciprofloxacin
		Essential oils		
		*C. validis*	*C. plurinodis*	
*Bacillus cereus*	G+	15.6	62.5	7.8
*Enterococcus faecalis*	G+	62.5	31.2	62.5
*Listeria monocytogenes*	G+	15.6	15.6	7.8
*Staphylococcus aureus*	G+	15.6	125.0	15.6
*Escherichia coli*	G-	62.5	500.0	15.6
*Klebsiella pneumonia*	G-	500.0	500.0	31.2
*Salmonella typhimurium*	G-	125.0	250.0	15.6
*Pseudomonas aeroginosa*	G-	500.0	>500.0	125.0

Both plant essential oils had little or no effect against the Gram-negative *Pseudomonas aeroginosa* due to its high level of intrinsic outer membrane barrier that is resistant to virtually all known antimicrobials and antibiotics [30]. Moreover, the results obtained are of a great importance particularly in the case of *Bacillus cereus*, *Staphylococcus aureus* and *Listeria monocytogenes*, which are well known for being resistant to a number of phytochemical compounds [31, 32]. Plant essential oils and extracts have been used for many thousands of years [33], especially in food preservation, pharmaceuticals, alternative medicine and natural therapies [30, 31]). Essential oils extracted from *C. citratus*, *C. flexosus*, *C. naudus* and *C. winterianus* exhibited activity against both Gram positive and Gram negative bacteria [34, 35]. Application of these essential oils on bacteria strains inhibited *Acinetobacter baumanii*, *Enterococcus faecalis*, *Escherichia coli*, *Klebsiella pneumonia*, *Pseudomons aeruginosa*, *Salmonella typhimurium*, *Serratia marcescens* and *Staphylococcus aureus* at the concentration of 1200 μg/ml to <20,000 μg/ml [36]. It has long been acknowledged that some plant essential oils exhibit antimicrobial properties, due to their monoterpene hydrocarbons, aldehydes and oxygenated monoterpenes. Compounds from these groups, such as Citronella, Camphene, Limonene, Sabinene, Geraniol and Phytol have been reported that they can diffuse into and damage cell membrane structures of organisms [37, 26].

### Cytotoxicological effect of the extracts on cell viability

The liver is known as a unique organ and primary site of detoxification. Due to the important role of the liver intense metabolism, it is likely to be prone to various disorders as a result of toxic chemicals [38]. Liver maintains the energy level and structural stability of the body. Therefore, any attempt to damage the liver either by any poisonous or harmful chemicals will definitely result to hepatotoxicity [38]. The toxicity of both plants extracts were tested to evaluate their effects on human Chang liver cells.

According to our cytotoxicity evaluation of both plants, we observed that at 5, 25, 50, 75, and 95 percentile of cell death after 24, 48, and 72 h of incubation, the *C. validis* extract dose activity ranged from log of 0.62 ± 0.03 to 0.99 ± 0.01, 0.76 ± 0.03 to 1.67 ± 0.01, 0.85 ± 0.02 to 2.09 ± 0.00, 2.05 ± 0.01 to 2.77 ± 0.01, and 3.45 ± 0.01 to 3.03 ± 0.01, respectively (Table 5). *Cymbopogon plurinodis* extract dose activity ranged from log of 0.74 ± 0.03 to 0.18 ± 0.01, 0.86 ± 0.03 to 1.80 ± 0.01, 0.18 ± 0.02 to 2.11 ± 0.00, 2.99 ± 0.01 to 2.45 ± 0.01, 3.95 ± 0.01 to 3.33 ± 0.01, respectively. Lethal dose of *C. validis* at 50 (LD_50_) showed 74.87 ± 1.41, 139.07 ± 1.29, and 122.06 ± 0.89 μg/ml degree of toxicity at 24, 48, and 72 h, respectively (Table 6). Lethal dose of *C. plurinodis* at 50 (LD_50_) showed 81.66 ± 1.40, 135.09 ± 1.30, and 120.02 ± 0.90 μg/ml degree of toxicity at 24, 48, and 72 h, respectively (Table 6).

**Table 5:** Effective concentration and time interval on the percentage cell death

*Cymbopogon validis*							*Cymbopogon plurinodis*						
	Probit			Log (dose)^a^				Probit			Log (dose)^a^		
Percentile	24 h	48 h	72 h	24 h	48 h	72 h	Percentile	24 h	48 h	72 h	24 h	48 h	72 h
5	2.89	2.89	2.89	0.62 ± 0.03	0.67 ± 0.01	0.99 ± 0.01	5	2.11	2.11	2.11	0.74 ± 0.03	0.60 ± 0.01	0.18 ± 0.01
25	3.67	3.67	3.67	0.76 ± 0.03	0.91 ± 0.02	1.67 ± 0.01	25	3.18	3.18	3.18	0.86 ± 0.03	0.77 ± 0.02	1.80 ± 0.01
50	4.88	4.88	4.88	0.85 ± 0.02	1.88 ± 0.01	2.09 ± 0.00	50	4.01	4.01	4.01	0.18 ± 0.02	1.69 ± 0.01	2.11 ± 0.00
75	5.13	5.13	5.13	2.05 ± 0.01	2.35 ± 0.00	2.77 ± 0.01	75	5.22	5.22	5.22	2.99 ± 0.01	2.75 ± 0.00	2.45 ± 0.01
95	6.23	6.23	6.23	3.45 ± 0.01	3.64 ± 0.01	3.03 ± 0.01	95	6.45	6.45	6.45	3.95 ± 0.01	3.58 ± 0.01	3.33 ± 0.01

^a^Antilog which gives lethal dose in μg/ml. Probit analysis NCSS 2007 used to determine log (dose), percentile and probit values

**Table 6:** Lethal dose at 50% reduction of the cell population

*Cymbopogon validis*										*Cymbopogon plurinodis*									
	Probit percent			Actual percent^a^			LD_50_ (μg/ml)^a^				Probit percent			Actual percent^a^			LD_50_ (μg/ml)^a^		
Dose (μg/ml)	24 h	48 h	72 h	24 h	48 h	72 h	24 h	48 h	72 h	Dose (μg/ml)	24 h	48 h	72 h	24 h	48 h	72 h	24 h	48 h	72 h
5	23.44	5.43	1.67	18.34	6.19	4.97	na	na	na	5	19.04	7.41	6.71	22.02	4.10	11.36	na	na	na
25	29.59	20.82	13.88	30.33	13.67	5.43	na	na	na	25	22.15	26.16	30.14	29.24	16.33	17.03	na	na	na
75	37.66	39.05	39.01	35.87	29.01	18.23	74.87 ± 1.41	na	na	75	32.06	41.55	42.16	41.11	24.91	23.20	81.66 ± 1.40	na	na
125	46.91	45.78	51.97	50.90	61.45	69.86	na	139.07 ± 1.29	122.06 ± 0.89	125	44.22	47.13	58.22	48.43	52.25	66.76	na	135.09 ± 1.30	120.02 ± 0.90
250	53.04	64.90	70.01	62.22	76.18	72.41	na	na	na	250	53.00	69.20	75.00	53.71	66.02	77.06	na	na	na
500	65.53	78.43	84.76	67.90	78.14	79.45	na	na	na	500	67.13	80.55	87.62	57.94	69.00	81.15	na	na	na
1000	78.33	88.34	90.55	74.48	80.69	83.82	na	na	na	1000	71.01	84.46	90.68	65.59	76.19	86.26	na	na	na

^a^LD_50_ (50% of the cells have been killed); actual and probit percents were calculated using probit statistical analysis software “NCSS 2007”; ^a^ actual % = actual formulas (n is the number of cells in a group); *na* not applicable

We also observed that there is no information in the literature on the microbial and cytotoxic effect of both plants leaves extracts. The use of cell-based screening assays has proven more relevant in predicting response of organisms to drug effect [39]. More also, evaluating cellular toxicity on the eukaryotic cell culture has been recognized as the model system of choice to get an approximation of toxicity [40]. The LD_50_ (lethal dose, 50%) indicates the quantity of extracts/compounds that, if administered to a population of organisms, will cause 50% of the organisms to perish. A high LD_50_ implies it would take a large quantity of the extract to cause a toxic response, while small LD_50_ values are highly toxic and could be dangerous. It was observed that the dose of both plants extracts appeared to be more toxic after 24 h. *Cymbopogon validis* at 24 h (log 0.85 ± 0.02; LD_50_ = 74.87 ± 1.41), was found more toxic than the treatment at 48 h (log 1.88 ± 0.01; LD_50_ = 139.07 ± 1.29) and 72 h (log 2.09 ± 0.00; LD_50_ = 122.06 ± 0.89). *Cymbopogon plurinodis* at 24 h (log 0.18 ± 0.02; LD_50_ = 81.66 ± 1.40) was also found more toxic than the treatment at 48 h (log 1.69 ± 0.01; LD_50_ = 135.09 ± 1.30) and 72 h (log 2.11 ± 0.00; LD_50_ = 120.02 ± 0.90). Cell-based lethality assay is an indication of cytotoxicity, bactericidal and various pharmacologic actions. The LD_50_ values obtained in the current study indicates that the plants extracts have high pharmacological activities [41, 40].

**Fig. 2:**
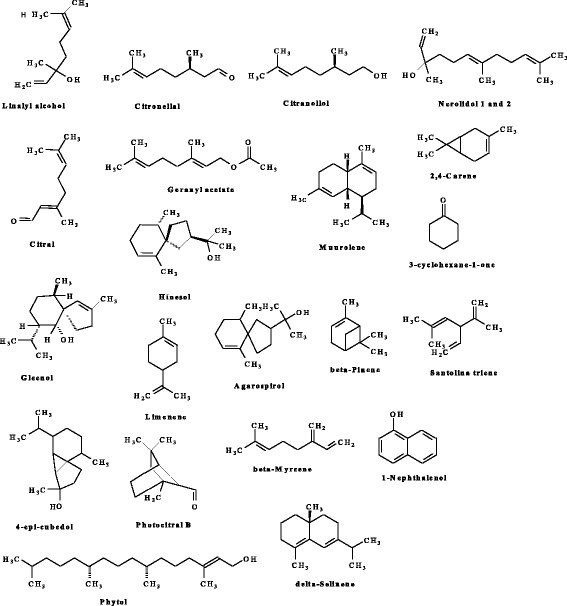
Some of the identified phyto-compounds that possesses antibacterial activity

The activities observed against bacterial strains and the human Chang liver cell may be ascribed to the phyto-compounds identified in both plants extracts. For example, inhaling linalool has been said to reduce stress-elevated level of neutrophils and lymphocytes in laboratory rats [42]. Citronellal, Citral, Geraniol, Geranyl acetate, Muurolene, Gleenol, Hinesol, Agarospirol, 4-epi-cubedol, Carveol, Photocitral B, Cubedol, Phytol, Epiglobulol, 2,4-Carene and Nerolidol are analgestic that can help reduce pain from strenuous activities and athletics, as well as toothaches, headaches, cough, cold, influenza, fever, and various poxes and inflammation, which can lead to many chronic diseases [27, 43, 44, 28] Fig. 2.

## Conclusion

The extracts of *C. validis* and *C. plurinodis* exhibited in-vitro antibacterial (both Gram-negative and positive species) activity. The major phyto-compounds revealed by GC-MS analysis are believed to be responsible for the antibacterial activity. However, since both plants extracts were toxic to the human Chang liver cells, we recommend that these plants extracts should be used with caution, and further studies using in-vivo (animal model) approach should be conducted to confirm this finding.

## Abbreviations

ANOVA: Analysis of variance
ATCC: America type culture collection
CFVR: Coronary flow velocity reserve
GC-MS: Gas chromatography-mass spectrometry
MIC: Minimum inhibitory concentration
MSD: Mass selective detector
